# Private Equity and Plastic Surgery

**DOI:** 10.1093/asj/sjad360

**Published:** 2023-12-14

**Authors:** Robert Singer

Mergers and acquisitions of medical practices following a private equity (PE) model have been a trend for other specialties (dermatology, ophthalmology, orthopedics), not always with a positive outcome.^1,2^ Plastic surgery and accompanying medical spas are currently the “plat du jour” for PE, and increasingly practices are being approached to sell their practices to PE.^3-7^ Plastic surgeons are often overwhelmed and do not know the pros and cons of joining these business ventures.

## CONS OF PRIVATE EQUITY

Historically, PE directors have a reputation for being very aggressive and self interested. Most of the PE entities know business, but not plastic surgery. They are predominantly interested in the financial bottom line, not patient service, and they often are not inclined to listen to the surgeons who know how to run a successful practice.^5^ Theoretically this model may make sense for those looking for a more balanced lifestyle, or an exit strategy, and for those tired of the rigors of running a practice with increasing expenses, ever more complicated regulations, and, if they accept insurance, decreasing reimbursements. But it is not for everyone. Certain personality types are not well suited for employee status or the loss of autonomy in making policy, running the practice, and hiring staff.^8^ Medical professionals and their potential business partners may and usually have differing agendas. Most of the leadership running PE come from banking and finance and are focused solely on the bottom line, treat plastic surgery no different from selling widgets, do not really care about patient safety, and often do not value physicians or staff.

## PROS OF PRIVATE EQUITY

Acquisition by PE might be reasonable as an exit strategy, as long as you are well aware of what you want and what you are getting into. It may allow you to continue to work without all the obligations of marketing, human resources, accounting, and information technology. It may also bring improved office efficiency—although often what occurs is not what is promised. There can and should be an economy of scale for supplies, equipment, implants, fillers, and neurotoxins if the business is structured correctly. There is a greater value of EBITDA (earnings before interest, taxes, depreciation, and amortization) if there are multiple practices vs 1 practice, but there also is dilution of your equity percentage as additional practices are brought in. Key factors to consider include: How long do you plan to practice? Can you accept giving up control?

If you are wondering if you have heard or seen this story in the past, you are correct. Over 2 decades ago, the Plastic Surgery Co. purchased plastic surgery practices with a financial model of cash and shares of stock.^9^ This venture went bankrupt and member surgeons had to buy back their practices from the bankruptcy court.

## STRUCTURE OF THE PRIVATE EQUITY ENTITY

There are numerous aspects to consider with regard to the structure of the PE entity. What is the economic stability of the entity and their track record? Is it a franchise model or does the individual practice exist under a bigger umbrella? What does the hierarchy of the organization look like and to whom will you and your staff report? Most private equity entities have an exit plan of 5 to 7 years, at which time they plan to flip the business, selling it to another entity or making a public offering. If they sell it, who will run it and what would be the philosophy of the next group? What happens to the individual practices and who will have oversight? Of key importance, will they continue the philosophy and culture of your practice, or will they change it? Essentially, you will have no control of the sale to a second group.

## FURTHER CONSIDERATIONS: TRACK RECORD, OVERSIGHT, REPORTING, COMPLIANCE, AND MORE

There are other considerations. Are you the first practice in the entity or is it already functioning, making you an added practice? If you are the initial practice, they may not have overcome the inefficiencies and obstacles that initially arise to make it run more efficiently. How will you know things are progressing if reports of finances both for the entire entity and for your practice are not provided on a timely basis? Various states have different corporate practices of medicine (CPOM) doctrines; does the PE entity comply with those and with Stark laws?^10^

Generally, firms have no idea about medical care and patient service, so ideally the entity should have a physician board. If they do, how much power does that physician board really have in decisions made or the direction the PE goes? Although these physicians typically have authority over all medical decisions, the PE partners can exert behavioral influences on physicians that can be ethically problematic because their basic concern is not patient service, it is growth and profits.

## VALUATION AND FINANCIAL CONCERNS

Before entering into an arrangement of sale, a key consideration is how long you want to keep working. Is this an exit strategy, or do you want to work longer? Will you be obligated to work a longer number of years, usually 5+ years? It is important for you to consider options related to the percentage of cash you receive up front vs the percentage of equity, which is often negotiable. It is absolutely essential to get legal advice from someone familiar with merger and acquisition arrangements before entering into a business relationship, as well as to have an established outside professional entity value your practice. You have to understand that the value of your own practice is not as great as you think, and that it depends on not only yearly income but on whether you own the building or the office suite. Is there a med spa? Do you have an accredited operating room? And if you do, how many rooms are there for surgery?

The valuation is one thing, but taxation of the sale is unique to each practice, and what will be most important to a business owner are the post-tax proceeds. To understand and maximize your benefits, consultation with an accountant or tax professional to ensure you, as the seller, understand the specifics based on your practice is vital. There are items to be incorporated in the contract, including an option to keep working should you choose to do so, a contractual clause of dismissal only for cause, and a stipulation that you have “x” dollars for medical organization fees, travel to meetings, registration fees, personal vehicles, etc; otherwise you may lose these pre-tax benefits. Other essentials include a disability plan, liability insurance for the office, and, because you will not practice forever, when you leave there needs to be tail-end malpractice coverage. Many of us have advisory positions in other ventures and there should be a carve out for whatever fees are brought in from those ventures. Remember that in any sale you are buying into this other entity. The devil is in the details; buyer beware—you only get to sell your practice once.

## CHANGES THAT MAY OCCUR

PE generally desires to bring on more practices enabling the entity to grow. What say, if any, do you have in vetting the new practices that are being considered, and how will they affect your image and reputation? What if there is a practice they are considering that has a questionable reputation? Within your own practice, how much say will you have when it comes hiring, firing, and personnel compensation? As ever, keep in mind that PE's concern is the bottom line, which is improved by more income or by cutting costs. Will those decisions be at your discretion, or will they be made by their corporate human resources personnel, who may not have adequate knowledge about your practice?

New systems of accounting, electronic medical records, and information technology may be brought in, with a learning curve for everyone in the office. Remember that change brings anxiety to you and your staff. What will be the learning curve with these new systems? Will they be easy to use? New policies with regard to staff receiving procedures, fillers, and neurotoxins may affect morale.

To continue a productive practice, it is often important to bring in new associates. What options exist for that within the entity? Will PE help recruit associates? Are there accommodations for equity for that younger associate? Will you have the ultimate say in the interview and hiring process?

## COMPENSATION

Compensation is always a concern, as is how it is calculated. Is it based on a salary plus a percentage of the billing, straight salary, or only on percentage of productivity? Do you have to reach a monthly production quota to get that compensation? Although the value of your equity may increase, it is possible your take-home pay may decrease.

Part of running a practice also consists of satisfying the patients, and sometimes there are revisions or unexpected sequelae in the immediate postoperative period that require return to the operating room. How will the cost of those be handled? Newer technologies come along that may benefit a practice. Will you have the final say on bringing in new equipment, lasers, injectables? Delays caused by waiting for corporate approval when ordering new supplies can hamper day-to-day operations.

## MARKETING

Marketing is a primary concern in most practices. Will the marketing be “branding” for the broader corporate PE entity or individually for your practice, and who will have control? Will social media as well as other marketing be performed at the corporate level or in-house by the individual practice? Marketing costs money. How much will be directed toward your advertising budget? Who will own your new website?

## CONTINGENCY PLANNING

Unfortunately, not every entity like this has been successful. If you become disgruntled, how difficult and expensive will it be for you to extricate yourself from the PE agreements? To get the value of your equity, do you have to wait for them to flip it or bring it public? There is generally a lack of liquidity. Also, if you do decide to terminate your employment and there is deferred compensation, you may forfeit this amount. You also may have a noncompete clause if you leave, although these have become increasingly unenforceable in many states.

## THE BOTTOM LINE

The PE concept is good and there will continue to be more and better PE firms entering into the plastic surgery arena, but success will depend on appropriate oversight and administration of the entity. Many plastic surgeons are accustomed to total control of their practices and would not comfortably fit into this business model because they would be loath to cede control and follow specific policies and rules. Although some PE firms insist on completely taking over and view the physicians as purely employees, the more successful PE firms are aware of the risks and are more interested in involving practice owners as physician leaders and directing growth from the sidelines.
